# Potential of the Ethyl Acetate Fraction of *Padina boergesenii* as a Natural UV Filter in Sunscreen Cream Formulation

**DOI:** 10.3390/life13010239

**Published:** 2023-01-14

**Authors:** Soolmaz Soleimani, Morteza Yousefzadi, Sepideh Babaei Mahani Nezhad, Olga N. Pozharitskaya, Alexander N. Shikov

**Affiliations:** 1 Department of Marine Biology, Faculty of Marine Science and Technology, University of Hormozgan, Bandar Abbas 7916193145, Iran; 2 Department of Biology, Faculty of Science, University of Qom, Qom 3716146611, Iran; 3 Murmansk Marine Biological Institute of the Russian Academy of Sciences (MMBI RAS), Vladimirskaya, 17, 183010 Murmansk, Russia; 4 Department of Technology of Pharmaceutical Formulations, St. Petersburg State Chemical Pharmaceutical University, Prof. Popov, 14, 197376 Saint-Petersburg, Russia

**Keywords:** brown seaweed, marine natural products, polyphenol compounds, sunscreen cream, cosmetics

## Abstract

Brown seaweeds, due to their wide range of bioactive compounds, have a high ability to inhibit free radicals and protect against ultraviolet rays. In the present study, the ethyl acetate fraction (EF) was isolated from the *Padina boergesenii* brown seaweed. Antioxidant activity (by the DPPH scavenging activity method) and cytotoxicity against UVB-induced cytotoxicity in HaCaT human keratinocytes were evaluated. Then, this fraction was used as a bio-filter in the formulation of sunscreen, and the physical properties and stability were investigated. The results showed that the EF could inhibit DPPH radical scavenging (54 ± 1%) and cell viability of HaCaT keratinocytes exposed to UVB irradiation (81.2 ± 0.1%). The results of the stability study of the cream formulated with EF showed that at temperatures 4 °C and 25 °C it has high stability; and at 40 °C on the 28th day, a slight decrease in its stability was observed. The pH and Sun Protection Factor of the cream formulated with EF were reported at 5.8 and 20.55, respectively. Also, the DPPH scavenging activity of the cream was not altered for 28 days of storage at temperatures of 4–40 °C. According to our results, it was proved that the sunscreen formulated with EF of *P. boergesenii* brown seaweed has promising properties and characteristics that can create a new opportunity for the development of cosmetics and skin care products.

## 1. Introduction

The epidermis of the skin, as the first barrier to the harmful effects of the environment, contains a variety of adaptive processes that are crucial for sun protection [1]. Sunlight is formed from wavelengths of the ultraviolet ray to visible light. The three types of ultraviolet rays are UVA (320–400 nm), UVB (290–320 nm), and UVC (100–290 nm) [2]. Immunosuppression, early aging, melanoma, and skin cancer are all prominent effects of UVB exposure in humans [3]. Inhibition of the skin epidermis’s ability to act as an antioxidant, as well as oxidative stress and free radical photodynamics, are the ways that UVB causes skin disorders [4]. Today, due to the increased mortality from skin cancer, preventive solutions must be found [5]. Sunscreen use and antioxidant supplements, whether taken orally or topically, are suggested ways to lower the risk of skin cancer [6,7]. Therefore, the production of natural products represents an alternative method for skin care [8,9].

Injurious conditions brought on by ultraviolet radiation include the production of free radicals and DNA damage in the skin [10]. Polyphenols are one of the many substances that plants produce to defend themselves from sunlight [11]. The production of polyphenols in plants largely depends on environmental conditions and exposure to sunlight [12,13]. Polyphenols have aromatic rings with one or more hydroxyl substituents as the foundation of their structure [14]. The UVB spectrum, as well as a section of the UVA and UVC spectrum, can be absorbed by these compounds [15]. Brown seaweed contains phloroglucinol units as part of its polyphenolic compounds. These compounds are created during growth as essential elements of the seaweed cell wall or as a chemical defense mechanism in response to stressful situations like UV radiation or bacterial infection [16]. Brown seaweed develops phloroglucinol-based polyphenols over time, which enhances its antioxidant activity against oxidative damage [17]. Antioxidant activity of brown seaweed such as *Padina australis* [18], *Padina pavonica* [19], *Sargassum horneri* [20], *Sargassum angustifolium* [21], *Cystoseria myrica* [21], *Eisenia bicyclis* [22], *Kjellmaniella crassifolia* [23], *Alaria crassifolia* [24], and *Cystoseira hakodatensis* [25] has been reported. Due to polyphenol compounds, brown seaweed has other biological activities such as antibacterial [26], antiviral [27], anti-cancer [28], anti-inflammatory [29], anti-allergic [30], and anti-aging [31]. Therefore, adopting seaweed as a source of natural ingredients may serve as an incentive for the development of a new cosmetics industry.

Given these facts, this study aimed to isolate the EF rich in polyphenol compounds from the brown seaweed *Padina boergesenii*. The ability of the EF to inhibit DPPH *(2,2-diphenyl-1-picrylhydrazyl)* free radicals and its protective effect against UVB on human keratinocytes was evaluated. Then, the EF was used in a sunscreen formulation. The organoleptic properties and stability of the cream formulation were investigated. 

## 2. Materials and Methods

### 2.1. Material

Analytical-grade chemicals and solvents for extraction were purchased from local chemical suppliers. Ascorbic acid, gallic acid, and 1,1-diphenyl-2-picrylhydrazyl were from Sigma-Aldrich, Darmstadt, Germany. All other specialized chemicals were obtained from Merck (Darmstadt, Germany). Deionized water filtered through a 0.22-μm filter was used for reagents and cream preparation.

### 2.2. Collection of Seaweed and Preparation of Fraction

In the intertidal zone of Qeshm Island (56°16′08′′ E, 26°56′57′′ N), Hormozgan Province, Iran, *P. boergesenii* seaweed was collected. Samples were collected, immediately brought to the lab, gently rinsed with filtered seawater, and then allowed to air dry for seven days at a temperature of about 25 °C.

**Figure 1:**
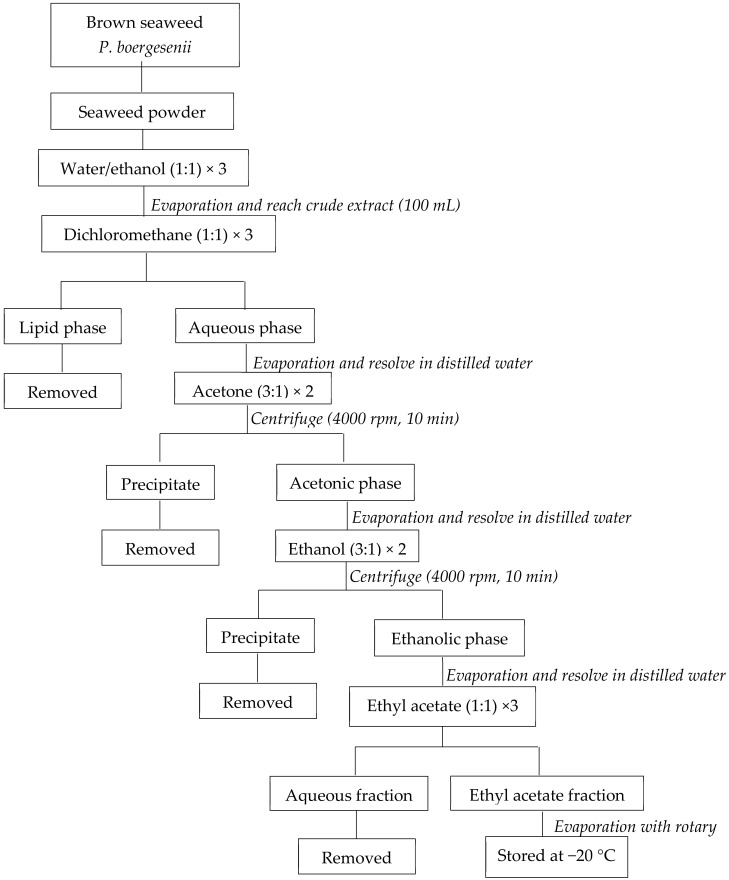
Preparation of *P. boergesenii* EF by liquid/liquid purification. adapted from Le Lann et al., 2016 [32].

The initial 30 g of dried seaweed powder and 300 mL of the water/ethanol mixture were mixed in the dark to extract EF from *P. boergesenii*. Under vacuum, the supernatant was evaporated to a volume of 100 mL. The mixture was subsequently rinsed with the corresponding amounts of dichloromethane, acetone, ethanol, and ethyl acetate. In the end, the EF evaporated under a vacuum and was kept at −20 °C (Figure 1) [32].

### 2.3. Determination of Total Phenol in P. boergesenii Fraction

Using the Folin-Ciocalteu method, the quantities of phenolic compounds in the prepared fraction were calculated [33]. The first involved combining 0.75 mL of Folin-Ciocalteu solution with 0.15 mL of EF at a 10 mg/mL concentration (diluted 1:10 with distilled water). Then, 0.6 mL of saturated 7.5% Na_2_CO_3_ (*w*/*v*) was added to the mixture after 3 min. The sample was mixed and left in the dark for one hour, and the absorbance was measured at 765 nm. Gallic acid’s standard curve (0.003–0.1 mg/mL) was used to calculate the total phenol concentration, and the results were represented in mg gallic acid·g^−1^ of dried weight of pure seaweed (DW).

### 2.4. Determination of DPPH Free-Radical Scavenging

The DPPH method was used to assess the EF’s free radical scavenging capacity [34]. At concentrations of 25, 50, 100, 200, and 300 µg/mL, the EF was put to the test. 100 μL of DPPH solution could react with 100 μL of the EF (0.5 mM). The materials were mixed and placed in the dark for 30 min, and the absorbance at 517 nm was gauged. Instead of the fraction, the control uses pure water, and the blank uses methanol rather than a DPPH solution. Formula 1 was used to determine the fractions’ DPPH scavenging activity.

DPPH scavenging activity (%) = [1 − ((A_s_ − A_0_)/A)] × 100 (1)

where A_s_ was the absorbance of the reaction mixture, A_0_ was the absorbance of the blank, and A was the absorbance of the control. Ascorbic acid (Vitamin C, Vit C) was used as a positive control.

### 2.5. Determination of UVB Irradiation Effect on the Human Keratinocyte Cell 

The Pasteur Institute of Iran provided the human keratinocyte HaCaT line for purchase. Fetal bovine serum (FBS, 10%) and penicillin-streptomycin (1%) were added to DMEM, and cells were grown at 37 °C in a CO_2_ incubator with 5% moisture.

The HaCaT cells were seeded at a density of approximately 1 × 10^5^ cells in order to assess the UVB protective abilities of *P. boergesenii*’s EF. After 24 h of adhesion-promoting incubation at 37 °C and 5% CO_2_, the cells were then examined. PBS and distilled water were used to dilute the fraction such that it could be tested at concentrations of 25, 50, 100, 200, and 300 µg/mL. Only distilled water and PBS were used to treat the aluminum foil-covered, unexposed control cells. Rosmarinic acid (RosA) was used as a positive control. After 24 h, the cells were concurrently concurrently exposed to 50 µL of each fraction concentration with 50 mJ/cm^2^ of UVB light. PBS was withdrawn from the cells after UVB irradiation, and they were then placed in 200 µL of brand-new growth media and incubated for 24 h at 37 °C in 5% CO_2_. After the initial incubation period, each well received 10 µL of MTT solution (5 mg/mL), which was then incubated for an additional 4 h at 37 °C. A gentle aspiration was used to remove the medium, 100 µL of distilled water was added to the formazan solution, and an ELISA reader was used to determine how much formazan salt was present. A UV-340A UV light meter was used to measure the strength of the radiation given to the cells by a Philips UVB Broadband TL 20 W/12 phototherapy lamp with wavelength bands 290 and 315 nm [35]. The following formula 2 was used to calculate the inhibition rate to assess the viability of the cells:

Cell viability (%) = (OD control − OD treatment)/(OD control) × 100(2)

### 2.6. Fourier Transform Infrared Spectroscopy (FTIR)

Specific chemical groups in the materials were characterized using FTIR. Semi-purified *P. boergesenii* was subjected to an FTIR examination spanning the wave-number range of 500–4000 cm^−1^ (Bruker Optics ALPHA-E Spectrometer with a universal Zn-Se ATR accessory).

### 2.7. Gas Chromatography and Mass Spectrometry Analysis (GC-MS)

A model 8000 Top GC outfitted with a Best PTV (programmed temperature vaporizer) injector, an AS800 autosampler, and a Voyager mass spectrometer was used for the GC-MS analysis (Interscience, Breda, The Netherlands). The MassLab software was used to operate the equipment. The injector had an inner surface made of porous sintered glass that was 1 mm in diameter. The GC was fitted with a pre-column of 2.5 m and a 30 m 0.25 mm i.d., 0.25 m film, HP-5-MS column (same as the analytical column, connected by means of a press-fit connector).

Then, 20 μL was injected at a rate of 5 μL s^−1^ for PTV injection in the solvent-vent mode. With a split flow of 100 mL per minute, the solvent was vented at 50 °C for 0.67 min. After that, the split valve was shut, and the analytes still present in the liner were transported to the GC column by ramping the temperature from 10° s^−1^ to 300 °C. The split was reopened after a 2.5 min total transfer time.

As a carrier gas, helium was employed at a continuous flow of 1.5 mL per minute. After the injection, the oven’s temperature was held at 90 °C for 2 min before being scheduled to rise to 300 °C and stay there for 10 min. At 305 °C, the transfer line to the MS was maintained.

At a source temperature of 200 °C, mass spectrometry was carried out using electron-impact (EI) ionization (electron energy 70 eV). After a solvent delay of 5.5 min, data were collected in full-scan mode (*m*/*z* 60–400) until 30 min. The scan time and inter-scan delay were 0.3 and 0.1 **s**, respectively, yielding 2.5 scans per second. The detector had a potential of 450 V.

For data handling and quantitative data evaluation, MassLab software (Interscience, Breda, The Netherlands) and an Excel macro developed in-house were used. To identify compounds, the NIST GC-MS library was used, and the closest match was recorded.

### 2.8. Preparation of Emulsions

The elements and concentrations of (W/O) emulsions are displayed in Table 1. Formulations F1, F2, and F3 were employed in this study. The phase A and phase B components at 70 °C were separately premixed until they formed a homogenous mass. Following that, phase A and phase B were progressively combined at 70 °C and swirled for 30 min. The base cream for the A and B phase mixtures was combined, and the phase C components were added. The basic cream received Phase D (negative control). Sterilization of the base cream and negative control was conducted for one hour at 60 °C. The negative control was then given 5% of the EF of *P. boergesenii*, and the mixture was agitated for one hour [36,37].

### 2.9. Determination of Stability of Formulated Creams

The stability test of the creams formulated was performed by two methods (cooling-heating cycle process, long-term stability) and parameters control: pH, colloidal stability, and organoleptic properties (color, odor, and shape of the visual cream). The test stability of the creams formulated was performed in a cooling-heating cycle process for 6 cycles. Each cycle comprised a 24 h storage process at 4 °C (refrigerator temperature), then 24 h storage at 45 °C [38]. The pH of the cream was assessed after each cycle.

Long-term stability for the creams formulated was performed at three different temperatures of 4 °C, 25 °C, and 40 °C for 28 days [39]. The shape, odor, liquefaction, and phase separation of all creams were observed visually after 28 days.

The stability of the formulated creams was performed by centrifugal tests after the preparation of the creams. The 10 g of each formulation was centrifuged at 5000 rpm and 25 °C for 10 min. Those tests were repeated after 1, 7, 14, 21, and 28 days of storage.

The pH value of the F3 formulation was measured at three different temperatures of 4 °C, 25 °C, and 40 °C after 1 day, 7 days, 14 days, 21 days, and 28 days of storage using a pH-meter (Mettler Toledo electrode, Switzerland).

### 2.10. Determination of Sun Protection Factor of Formulated Creams

The sun protection efficiency of the formulated creams was determined on day 1, day 3, day 7, day 14, day 21, and day 28. Specifically, 50 mg of each formulation was spread using a cot-coated finger across the entire surface on a plastic methyl methacrylate (PMMA) plate. Transmission measurements were performed between 200 and 400 nm using a spectrophotometer with an integrated sphere. Three plates were prepared for each cream and 6-time measurements were made on each plate. Also, the Sun Protection Factor (SPF) of formulation F3 was determined at temperatures of 4 °C, 25 °C, and 40 °C on day 1, day 3, day 7, day 14, day 21, and day 28. The value of SPF in laboratory conditions was obtained from the mean of UV transmission at each wavelength according to formula 3 [40]:

(3) $$\mathrm{SPF}=\frac{\int_{290}^{400}E\left(\lambda \right)I\left(\lambda \right)d\lambda }{\int_{290}^{400}E\left(\lambda \right)I\left(\lambda \right)T\left(\lambda \right)d\lambda }$$

where Eλ was the erythema action spectrum at λ, Iλ was the solar spectrum radiation at λ, and Tλ was the spectral transmission of the sample at λ.

### 2.11. Antioxidant Activity of the Formulated Cream Containing EF

The free radical scavenging activity of cream containing EF (F3) and base cream (F1). Immediately after preparation, ??and formulation F3 after storage at three different temperatures of 4 °C, 25 °C, and 40 °C for 28 days was evaluated using the DPPH method. To perform this test, 1 g of each formulation was dissolved separately in 1.5 mL of ethanol and mixed for 5 min using vortex, followed by a mixture for 10 min of sonication. The mixture was centrifuged at 7000 rpm at 25 °C for 10 min; then, the supernatant was collected and stored for antioxidant activity using a DPPH assay. Then, 100 μL of cream supernatant was mixed with 100 μL of DPPH solution (0.5 mM) and stored in the dark at room temperature for 30 min. The absorbance of the samples was measured at 517 nm and 600 nm (as a reference wavelength). The control contains ethanol instead of the supernatant of creams [41]. The DPPH scavenging activity by fractions was calculated using formula 4:

DPPH scavenging activity (%) = [(A_517_ control − A_600_ control) − (A_517_ Sample − A_600_ Sample)/(A_517_ control − A_600_ control)] × 100 (4)

### 2.12. Statistical Analysis

The data were expressed as the mean ± standard deviation of three replicates. The analysis was performed using SPSS 21 (Chicago, United States) and Excel 2019. One-way analysis of variance (ANOVA) and Duncan’s new multiple-rang test was used to determine the possible differences amongst the means. *p* values ≤ 0.05 were considered significant differences.

## 3. Results

### 3.1. Total Phenols in P. boergesenii Fraction

The yield of the EF from 30 g dry seaweed powder was 0.1 g. The total phenolic content of the EF isolated from *P. boergesenii* was 14 ± 0.1 mg GAE·g^−1^ DW.

### 3.2. Antioxidant Activity

**Figure 2:**
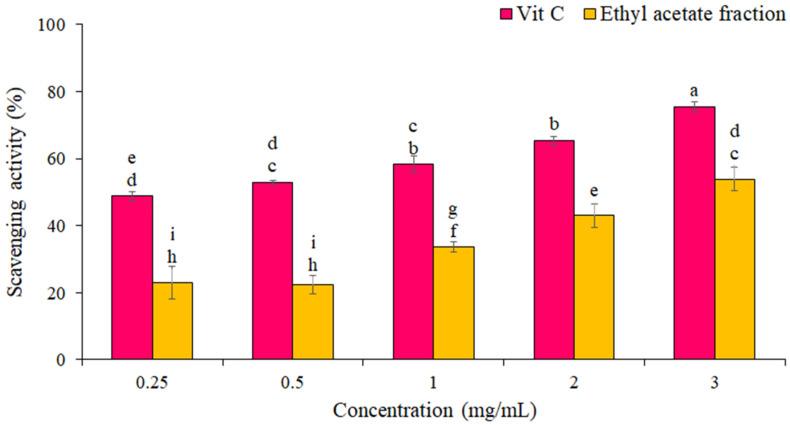
Percentage of DPPH radical scavenging by EF and Vitamin C. The data are the means and standard deviations (SD) of three independent experiments, each carried out in triplicate; means with different letters are significantly different.

The percentage of DPPH free radical scavenging by the EF from *P. boergesenii* compared to vitamin C as a standard is shown in Figure 2. The results showed that the scavenging of the DPPH free radical increased with increasing concentration. The highest scavenging of DPPH radicals by EF (54 ± 1 %) was at the concentration of 3 mg/mL. IC_50_ for the EF and vitamin C against scavenging of DPPH radicals were 2.6 ± 1.2 mg/mL and 0.24 ± 0.10 mg/mL, respectively. Statistical analyses showed significant differences in the DPPH free radical scavenging percentage between different concentrations (*p* ≤ 0.05). 

### 3.3. UVB Irradiation Effect on the Human Keratinocyte Cell 

**Figure 3:**
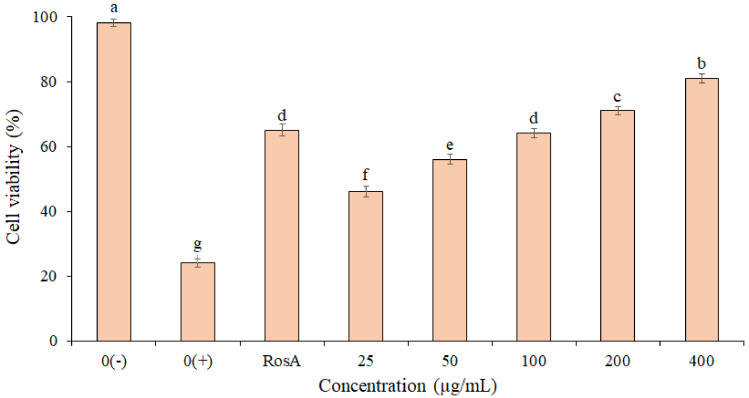
Protective effect of *P. boergesenii* EF against UVB-induced cytotoxicity in HaCaT keratinocytes. Distilled water was used to treat the negative (0+) and unexposed (0−) controls. The data are the means and standard deviations (SD) of three independent experiments, each carried out in triplicate; means with different letters are significantly different.

The photo-protective effect of the EF from P. boergesenii on HaCaT keratinocytes exposed to UVB Irradiation (50 mJ/cm^2^) is shown in Figure 3. The highest cell viability (81.2 ± 1.1%) was observed for cells treated with the EF at 400 µg/mL. Based on the results, the cell viability in the EF (concentrations 200 and 400 µg/mL) was higher than the RosA. There was no significant difference between the RosA and EF (concentration 100 µg/mL). These findings imply that the EF may have UVB protection properties. Statistical analyses revealed significant differences in the percentage of cell viability between concentrations (*p* ≤ 0.05).

### 3.4. FTIR Characterization of Ethyl-Acetate Fraction

**Figure 4:**
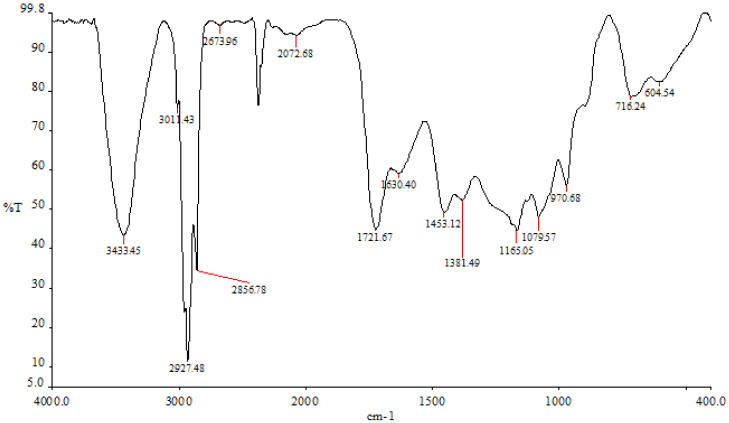
*P. boergesenii’s* ethyl-acetate fraction in the FTIR spectrum.

The findings demonstrated that various peaks in the FTIR spectrum were present when the ethyl-acetate fraction ranged between 3498 cm^−1^ and 720 cm^−1^ (Figure 4). The hydroxyl tensile vibration and hydrogen bonding are related to the peak at 3433 cm^−1^. Peak 2927 cm^−1^ is related to the CH-tensile vibration, which is a member of the family of methoxy chemicals. The C-C stretch of phenolic groups, which was measured in this study at 1453 cm^−1^, ranges from 1400 to 1500 cm^−1^ (corresponding to the aromatic nucleus). The stretching of C-O groups is related to the peak of 1381 cm^−1^. At a peak of 1165 cm^−1^, the sulfonate radicals first formed in the ethyl-acetate fraction. There are some carbohydrates present, as indicated by the peak at 1079 cm^−1^, which is caused by the vibrations of the glycosidic bonds C-O-C and C-O-H.

### 3.5. Gas Chromatography and Mass Spectrometry Analysis 

The GC-MS analysis was performed to identify compounds in *P. boergesenii*. The total ion chromatograph (TIC) corresponding to the compounds fractionated with EF from brown seaweed *P. boergesenii* is shown in Figure 1, and their peak identity is depicted in Table 2. Among the 17 identified compounds, five compounds with the highest area percentage and related retention time (Rt) are provided in Table 2. Based on the data of GC-MS phenol, 2-methyl-5-(1-methyl ethyl) was suggested as the main phenolic.

### 3.6. Properties and Stability of Formulated Creams

The organoleptic properties and stability of formulation creams were investigated during 28 days of storage at 4 °C, 25 °C, and 40 °C (Table 3). The results showed that the shape and odor of the formulated creams remained unchanged after storage at different temperatures for 28 days. The color of the creams freshly formulated was white for F1 and F2, and pale yellow for F3. The color of F1 and F2 formulations did not change after storage at 4 °C, 25 °C, and 40 °C for 28 days. The color of the F3 formulation remained unchanged at all three temperatures until day 21 of storage, while a slight change (yellowish white) was observed after day 21 of storage at 40 °C.

The results showed that no phase separation or liquefaction was observed for the formulated creams during 28 days of storage at temperatures of 4 °C, 25 °C, and 40 °C (Table 3). The results of centrifugal tests showed that no phase separation was observed in any of the formulated creams at 4 °C, 25 °C, and 40 °C during 21 days of storage, whereas after 21 days of storage, slight phase separation was observed in F2 and F3 formulations at 40 °C.

### 3.7. pH Value Determination

**Figure 5:**
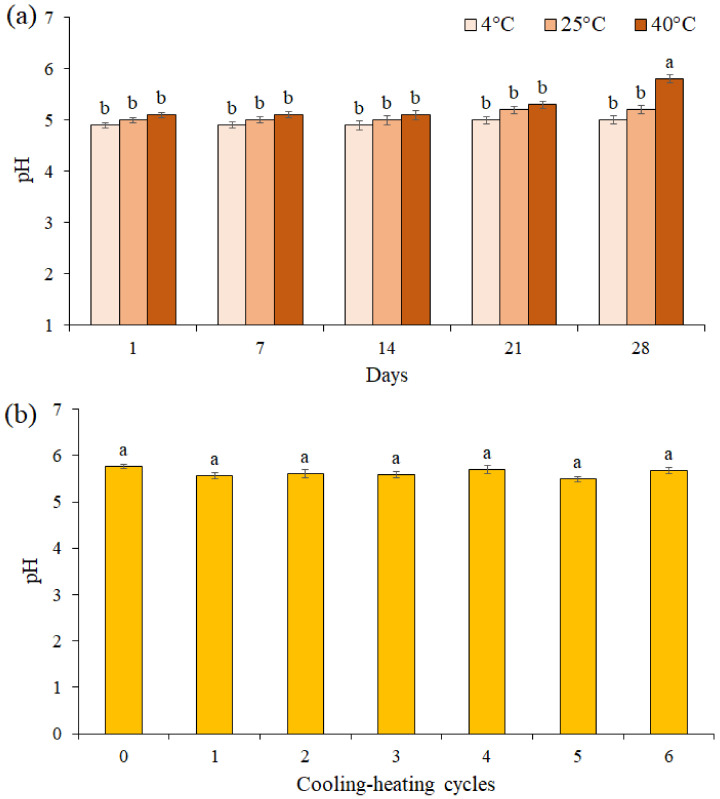
The effects of storage at different temperatures (**a**) and cooling-heating cycles (**b**) on pH of cream formulation F3. Data are means ± standard deviation (SD) of three independent experiments, each performed in triplicate. Significant differences are indicated by different letters as determined by Duncan’s Post-Hok multiple comparison (*p* < 0.05).

The pH value of the F3 formulation during 28 days of storage at different temperatures was presented in Figure 5a. The results showed the pH value was unchanged for up to 21 days of storage at different temperatures, while on the 28th day at 40 °C, a significant decrease was observed between pH values in the F3 formulation (pH = 5.8). A comparison of the pH values of the F3 formulation in 6 cooling-heating cycles did not show a significant difference between the pH values (*p* ≤ 0.05, Figure 5b).

### 3.8. SPF Value Determination

**Figure 6:**
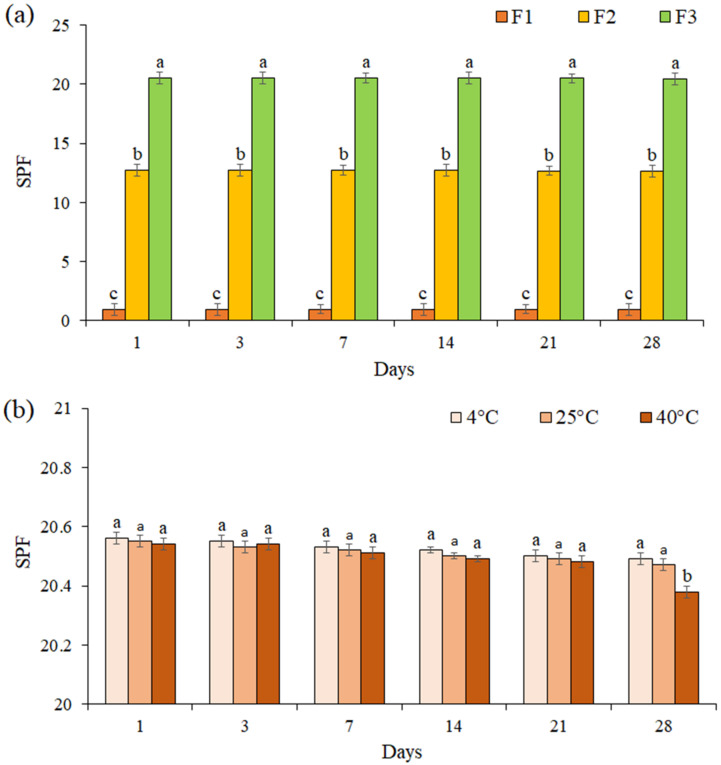
The SPF of formulations F1, F2, and F3 during 28 days (**a**), The SPF of formulation F3 at 4 °C, 25 °C, and 40 °C during 28 days of storage (**b**). Data are means ± standard deviation (SD) of three independent experiments, each performed in triplicate. Significant differences are indicated by different letters as determined by Duncan’s Post-Hok multiple comparison (*p* < 0.05).

The SPF for all formulations was evaluated for 28 days (Figure 6a). The results showed that the SPF of each formulation did not change significantly during 28 days (*p* ≤ 0.05). The SPF of cream containing EF (F3 formulation) was evaluated at 4 °C, 25 °C, and 40 °C during 28 days of storage (Figure 6b). We noted that the SPF of formulation F3 was not significantly changed at the different temperatures during 21 days (*p* ≤ 0.05), while on day 28 at 40 °C, a significant decrease was observed in the SPF (SPF = 20.38).

### 3.9. Evaluation of DPPH-Free Radical Scavenging Activity of Cream Containing EF after Storage

Table 4 presents the evaluation of DPPH free radical scavenging activity of cream containing EF and base cream immediately after preparation, as well as sunscreen formulation F3 after storage at the three temperatures of 4 °C, 25 °C, and 40 °C for 28 days. The results showed that the cream was stable at 4 °C, as well as 25 °C, after 28 days of incubation. On the other hand, the ability of formulation F3 to scavenge DPPH radicals after storage at 40 °C for 28 days was slightly reduced. There was no statistically significant difference in DPPH radical scavenging activity of formulation F3 during 28 days of storage at three temperatures (*p* ≤ 0.05).

## 4. Discussion

Phenolic compounds are known to protect against skin cancer due to their absorption of light [42]. These compounds are composed of aromatic rings with one or more hydroxyl groups [43]. The response of different extracts from seaweed in the Folin–Ciocalteu method depends on the number of phenolic groups in the seaweed [44]. Many studies have shown that polyphenolic compounds are abundant in extracts and fractions isolated from seaweed with polar and semi-polar solvents such as ethyl acetate and ethanol [45,46,47,48]. The phenolic content of the EF was reported from *Padina australis* (807.20 mg GAE·g^−1^) [18], *Padina tetrastromatica* (589.79 mg GAE·g^−1^) [49], *Salicornia ramosissima* (431 mg GAE·g^−1^) [11], *Halidrys siliquosa* (975.73 mg GAE·g^−1^) [32], *Turbinaria ornata* (69.63 mg GAE·g^−1^) [50], and *Turbinaria conoides* (105.97 mg GAE·g^−1^) [50]. Semi-pure fractions compared to pure compounds have several advantages, including high extraction efficiency, balancing the concentration of extracted active compounds, cost effectiveness for various industrial applications, including cosmetic formulations [51].

The main phenolic compounds identified in the EF from *P. boergesenii* by GC-MS are abundant for brown algae *Padina pavonica* and *Hormophysa triquetra* [52], *Padina boergesenii* and *Sargassum cinereum* [53], *Canistrocarpus cervicornis* [54], *Laminaria japonica* [55], *Cystoseira America*, and *Sargassum wightii* [56].

Prolonged exposure to ultraviolet rays causes oxidative stress, which leads to inflammation and premature aging of the skin [57]. Polyphenolic compounds as powerful antioxidants play an important role in scavenging free radicals and reducing oxidative stress [58]. The DPPH free radical scavenging activity of the EF of *P. boergesenii* was similar to that of phloroglucinol isolated from *Turbinaria conoides* [59]. The EF of *Nannochloropsis oculata* had 39.03 ± 0.97% at 400 µg/mL [60] and *Sargassum pallidum* had 30.50 ± 0.03% at 2 mg/mL [61] inhibition of DPPH.

UVB rays cause destructive effects, such as oxidative stress [62]. The findings of the present study showed that the EF could reduce UVB-induced cytotoxicity in HaCaT cells. The protective effect of UVB *P. boergesenii* is probably due to polyphenolic compounds [63]. Many studies have shown that polyphenolic compounds derived from brown seaweed increase the viability of human fibroblast cells exposed to UVB [63,64,65,66].

Nowadays More recently, researchers have found that the use of synthetic compounds in cosmetic formulations may cause side effects such as skin irritation and inflammation. Natural compounds derived from seaweed are a good alternative for the development of natural cosmetics due to their extensive bioactivities. Various cosmetics companies focus on natural products extracted from seaweed [67]. One of the best-selling products that used *Padina pavonica* extract in its formulation is Elemis Pro-Collagen Marine Cream, which in 2015 clinical trials proved to reduce fine lines (https://uk.elemis.com/pro-collagen-marine-cream.html (accessed on 25 March 2019). Nevertheless, the use of seaweed itself in cosmetics may cause skin problems, such as irritation and inflammation. Thus, the use of algal fractions/extracts in the cosmetical formulation has been proposed as an alternative. In this study, *P. boergesenii* brown seaweed EF was used as a biological filter in a sunscreen formulation. The use of W/O emulsions has received much attention in many research fields [68]. In the current study, the organoleptic properties of formulated cream with the EF of *P. boergesenii* brown seaweed during 28 days of storage at 4 °C, 25 °C, and 40 °C showed that its odor and shape remained unchanged but its color changed from pale yellow to yellowish white on day 28 (Table 3).

One characteristic of the cream that plays an important role in its consistency is viscosity [69]. Over time, as the temperature increases, the formulated cream begins to separate, which reduces the viscosity and consequently increases the liquefaction [70]. Creaming causes the separation of phases due to the difference in the density of the two phases under the influence of gravity [71]. This study showed that for the cream formulated during 28 days of storage at temperatures of 4 °C, 25 °C, and 40 °C, no liquefaction was observed. Also, in the formulated cream, after 21 days of storage at 40 °C, slight phase separation was observed in F2 and F3 formulations. This stability is probably due to the rate of homogenization during the cream formulation, which prevents the formulation from breaking during the test.

One of the important factors in determining the chemical stability of formulated creams is pH [72]. The pH changes indicate a chemical reaction that can indicate the final quality of the product. Typically, the pH of human skin varies in the range of 4.5–6 [73]. Therefore, if the pH of a formulation is in this range, it can be used in the cosmetics industry. The pH of the cream formulated with the EF of *P. boergesenii* brown seaweed in this study after 28 days of storage at different temperatures was 5.8, which is in the pH range of human skin. These results indicate that the formulated cream is stable and significantly suitable for the skin and does not irritate the skin.

Another important factor to demonstrate the effectiveness of a sunscreen formulation is SPF [74]. According to the FDA monograph, sunscreen products are divided into three categories based on the degree of UV protection: SPF 2–12 (low protection), SPF 12–30 (medium protection), and SPF > 30 (high protection) (http://www.sun-protection-and-products-guide.com/SPF.html (accessed on 12 November 2021)). SPF cream formulated with the EF of *P. boergesenii* brown seaweed in this study reported 20.55. Many studies have reported on the determination of SPF in sunscreens in various forms (creams, lotions, and gels) [75,76,77]. Although adding more brown algae fraction to a cream formulation may increase its protective properties, doing so could create an unstable emulsion. Our previous research revealed that the appearance and pH of cream that contained a 5% brown algae fraction were unaffected. However, most of the authors do not pay attention to the behavior of sunscreen when exposed to high temperatures. In this study, the F3 formulation was stored for 28 days at a temperature up to 40 °C. Notably, SPF = 20.38 was found on day 28 at 40 °C, which indicates the stability and moderate protection against the UV rays of the formulated cream. According to the results obtained in this study, we believe that the EF of *P. boergesenii* can be introduced to the cosmetics industry as a natural UV filter for use in the formulation of sunscreens.

Our findings demonstrated that the EF offered formulation F3 DPPH free radical scavenging activity. Notably, despite being stored for 28 days at 4–40 °C, this activity remained steady (Table 4). Our findings showed that the cream formulation we produced was stable.

## 5. Conclusions

In HaCaT human keratinocytes, the EF of brown seaweed from *P. boergesenii* demonstrated strong antioxidant properties against DPPH free radicals and protective benefits against UVB-induced cytotoxicity. The outcomes demonstrated that the cream’s pH was within the constrained pH range of human skin. The cream’s designed formulation offers SPF 20.55, which is a moderate level of sun protection. During the heating-cooling cycle, the cream remained steady. After being stored at temperatures between 4 and 40 °C for 28 days, the cream’s ability to remove DPPH was unaffected. These results allow the EF to be recommended as a stable bio-filter to the cosmetics and pharmaceutical industries.

## Figures and Tables

**Table 1:** Components and concentrations of the formulations.

Phase	Ingredients	Formulations (%)		
		F1	F2	F3
A	Glycerol and 1,2-Propanediol	27	27	27
A	Potassium hydroxide	6.63	6.63	6.63
A	Deionized water	Added to make mixture up to 100 g	Added to make mixture up to 100 g	Added to make mixture up to 100 g
B	Stearic acid	30	30	30
B	1-Hexadecanol	1	1	1
C	Potassium cocoyl glycinate	9	9	9
C	Distilled glycerin monostearate	1	1	1
D	Zinc oxide	0	2.5	2.5
E	Ethyl acetate fraction of *P. boergesenii*	0	0	5

**Table 2:** Specific identified chemical compounds in *P. boergesenii* by GC-MS analysis.

Compound Name	Rt (min)	Area (%)
Tetradecane 3,5-Octadiene, 4,5-diethyl	3.167 3.322	2.638 0.739
Hexadecane	4.708	3.575
Benzene, 1,4-bis(1,1-dimethylethyl)	5.2	2.639
3H-1,3,4-Benzodiazepin-2-one, 1,2-dihydro-3,5-dimethyl	5.376	0.803
Benzene, p-di-tert-pentyl	6.05	5.448
1-Azapyrene	6.259	2.100
Trispiro [2.1.2.1.2.1]dodecane-4,8,12-trione	6.617	16.053
2-Methyl-6,7-methylenedioxy-4[1H] quinolone	6.836	12.233
Indeno(1,2,3-ij)isoquinoline	6.949	1.651
10-Methyl-9-cyanoanthracene	7.082	4.031
Phenol, 2-methyl-5-(1-methylethyl)	7.136	1.793
10-(cyanomethylene)anthrone	7.639	3.579
2,3,5,6-tetramethyl-6-(3′-methylbuta-1′,2′-dienyl) cyclohexa-2,4-dien-1-one	8.419	1.889
Naphthalene, 1,2,3,4-tetrahydro-1-methyl-	8.874	6.396
6-methoxy-2,3,4,4a,5,10-hexahydrobenzo[g]quinolinium chloride	9.206	3.849
Bicyclo [4.2.0]oct-7-ene	12.763	0.056

**Table 3:** Organoleptic characteristics of F1, F2, and F3 formulations kept at different temperatures.

Parameters		Fresh			1 Day			7 Day			14 Day			21 Day			28 Day		
		F1	F2	F3	F1	F2	F3	F1	F2	F3	F1	F2	F3	F1	F2	F3	F1	F2	F3
Shape	4 °C 25 °C 40 °C	− − −	− − −	− − −	− − −	− − −	− − −	− − −	− − −	− − −	− − −	− − −	− − −	− − −	− − −	− − −	− − −	− − −	− − −
Odor	4 °C 25 °C 40 °C	− − −	− − −	− − −	− − −	− − −	− − −	− − −	− − −	− − −	− − −	− − −	− − −	− − −	− − −	− − −	− − −	− − −	− − −
Color	4 °C 25 °C 40 °C	W W W	W W W	PY PY PY	W W W	W W W	PY PY PY	W W W	W W W	PY PY PY	W W W	W W W	PY PY PY	W W W	W W W	PY PY PY	W W W	W W W	PY PY YW
Liquefaction	4 °C 25 °C 40 °C	− − −	− − −	− − −	− − −	− − −	− − −	− − −	− − −	− − −	− − −	− − −	− − −	− − −	− − −	− − −	− − −	− − −	− − −
Phase separation	4 °C 25 °C 40 °C	− − −	− − −	− − −	− − −	− − −	− − −	− − −	− − −	− − −	− − −	− − −	− − −	− − −	− − +	− − +	− − −	− − +	− − +
Centrifugation	4 °C 25 °C 40 °C	− − −	− − −	− − −	− − −	− − −	− − −	− − −	− − −	− − −	− − −	− − −	− − −	− − −	− − +	− − +	− − −	− − +	− − +

−: No change; +: Slight change; W: white; PY: Pale yellow; YW: Yellowish white.

**Table 4:** DPPH free radical scavenging activity of sunscreen formulation F3 after storage at different temperatures.

	0 Days of Storage		Formulation F3 after 28 Days of Storage		
Samples	F1	F3	4 °C	25 °C	40 °C
Scavenging activity (%)	5.3 ± 0.9	48.3 ± 1.3	46.9 ± 1.1	48.1 ± 1.5	41.2 ± 1.3

F1: base cream, F3: cream containing EF, Data are means ± standard deviation (SD) of three independent experiments, each performed in triplicate.

## Data Availability

All data generated or analyzed during this study are included in this published article.
